# Multicenter Evaluation of an ELISA for the Detection of *Cryptosporidium* spp. Antigen in Clinical Human Stool Samples

**DOI:** 10.3390/microorganisms9020209

**Published:** 2021-01-20

**Authors:** Romy Razakandrainibe, Célia Mérat, Nathalie Kapel, Marc Sautour, Karine Guyot, Gilles Gargala, Jean-Jacques Ballet, Patrice Le Pape, French Cryptosporidiosis Network, Frédéric Dalle, Loïc Favennec

**Affiliations:** 1 EA ESCAPE 7510, University of Medicine Pharmacy Rouen, 76000 Rouen, France; gilles.gargala@chu-rouen.fr (G.G.); balletjeanj@laposte.net (J.-J.B.); 2 CNR LE Cryptosporidiosis, Santé Publique France, 76000 Rouen, France; 3 Department of Parasitology/Mycology, University Hospital of Nantes, 44093 Nantes, France; celia.merat@chu-nantes.fr (C.M.); patrice.lepape@chu-nantes.fr (P.L.P.); 4 Department of Parasitology/Mycology, Faculty of Pharmacy, Paris University, 75013 Paris, France; nathalie.kapel@aphp.fr; 5 Department of Parasitology/Mycology, University Hospital of Dijon, 21000 Dijon, France; marc.sautour@chu-dijon.fr (M.S.); frederic.dalle@chu-dijon.fr (F.D.); 6 Department of Parasitology/Mycology, Lille Pasteur Institute, 59000 Lille, France; karine.guyot@pasteur-lille.fr; 7 CNR LE Cryptosporidiosis Collaborating Laboratory, Santé Publique France, 21000 Dijon, France

**Keywords:** cryptosporidiosis, *Cryptosporidium* spp., diagnosis, coproantigen ELISA, large-scale epidemiology

## Abstract

Human cryptosporidiosis remains underdiagnosed, and rapid/accurate diagnosis is of clinical importance. Diagnosis of the *Cryptosporidium* oocyst in stool samples by conventional microscopy is labor-intensive, time-consuming, and requires skillful experience. Thus, we aimed to evaluate the usefulness of a coproantigen enzyme-linked immunosorbent assay (ELISA) test in detecting *Cryptosporidium* spp. from fecal specimens. For this aim, we evaluated the performances of a commercial ELISA (CoproELISA *Cryptosporidium* kit, Savyon Diagnostics, Israel) for the detection of *Cryptosporidium* spp. in random clinical stool samples through a multicenter study. The sensitivity and specificity for coproantigen ELISA were 98.86% and 94.32%, respectively. The coproantigen ELISA results indicate that the simple, rapid, reliable, and standardized immunoassay test is sensitive and specific for routine diagnosis, and may be useful for large-scale epidemiological studies of cryptosporidiosis.

## 1. Introduction

Cryptosporidiosis, an emerging infectious disease of public health significance worldwide, is caused by the protozoan *Cryptosporidium*. Currently, twenty-three species and 61 valid genotypes of *Cryptosporidium* spp. have been described from a wide range of vertebrates, including humans, mammals, wildlife, domestic livestock, reptile, birds, amphibians, and fish, causing asymptomatic or mild-to-severe gastrointestinal disease in its host species [1].

*Cryptosporidium parvum* and the anthroponotic *Cryptosporidium hominis* are the major causes of human cryptosporidiosis. Although infection is asymptomatic, sometimes it causes diarrhea with a large number of oocysts present in the stools, as the acute infection resolves and the patient becomes asymptomatic, the number of oocysts decreases. *Cryptosporidium* can also cause chronic diarrhea, abdominal pain, weakness, weight loss, which can result in death in immunosuppressed individuals [2,3]. The diagnosis of cryptosporidiosis is usually made by microscopic detection of the parasite oocysts. However, this method is labor-intensive, time-consuming, requires skillful experience, and has low sensitivity [4,5]. Molecular biology techniques have become diagnostic tools that have been used to understand the epidemiology of *Cryptosporidium* spp. However, the accessibility to this technique is limited in some laboratories and totally absent in others. In addition, the expense and requirement for technical expertise have limited their use, particularly in high-prevalence regions, such as developing countries.

Enzyme immunoassay (EIA) for detecting antigen has been introduced successfully for *Cryptosporidium* screening in feces [5,6] Demonstration of cryptosporidial antigen in stool using ELISA is useful for screening large numbers of specimens. Several commercial immunochromatographic assays are currently available; however, previous comparisons have revealed limitations in their sensitivities according to *Cryptosporidium* species [7]. Potential advantages of commercially available ELISA kits for coproantigens are thus standardization of reagents, potential automatable process, and reproducibility in the context of administrative validation procedures. However discrepant data are available concerning this method [8].

The aim of the present study is to evaluate the performances of a commercial ELISA (CoproELISA *Cryptosporidium* kit, Savyon Diagnostics, Israel) for the detection of *Cryptosporidium* spp. in random clinical stool samples through a multicenter study.

## 2. Materials and Methods

The study was conducted in three medical parasitology laboratories from the University Hospitals of Rouen (Lab#1), Nantes (Lab#2), and Dijon (Lab#3) with recognized proficiency in the detection of *Cryptosporidium* spp.

Fifty stool samples containing *Cryptosporidium* oocysts were provided by the French *Cryptosporidium* National Network [9] and separately investigated by Lab#1 and Lab#2.

In addition, 12 and 28 fresh *C. parvum* oocyst-positive random samples were assayed separately by Lab#1 and Lab#3, respectively. The diagnosis was established by microscopy, and then the *Cryptosporidium* species determined by PCR sequencing at the 18S ribosomal DNA locus [10] which consisted of *C. parvum* (*n* = 20), *C. hominis* (*n* = 20), *C. felis* (*n* = 6), *C. meleagridis* (*n* = 2), *C. canis* (*n* = 1), and an *C. chipmunk* (*n* = 1). Each study center was also provided with 56 (Lab#1), 60 (Lab#2) and 60 (Lab#3) potassium dichromate fixed (K_2_Cr_2_O_7_ PBS) negative controls in which the absence of *Cryptosporidium* spp. was screened by microscopy. Secondly all negative controls were studied by PCR.

*Cyclospora* and *Cystoisospora* oocysts’ strong autofluorescence properties render fluorescence microscopy useful for identification [11]. In epifluorescence microscopic examination, using a 330–380-nm ultraviolet filter, *C. cayetanensis* oocysts can be easily visible in clinical samples [12]. Oocysts of *Cystoisospora* can be differentiated by their roundish appearance, their thin, smooth wall and, after sporulation, by the number of sporocysts [13]. None of the samples contained other Coccidia, i.e., *Cyclospora* or *Cystoisospora*.

To assess human clinical stool preservation’s effects on the CoproELISA performance, we used five fresh stool samples with *C. parvum* oocyst counts ranging from 4 to 76 per 50 microscopic fields (MF).

For each stool, four conditions were studied, i.e., undiluted stool, 1:10 stool dilution in PBS, 1:1 stool dilution in 2.5% potassium chromate (K2CRO4), PBS, and undiluted stool kept frozen at (−80 °C) for ≥48 h.

In each laboratory, ELISA were performed in triplicates according to the manufacturer’s instructions (Savyon Diagnostics, Ashrod, Israel) by the same experienced staff member to minimize handling risks and reading errors. The *Cryptosporidium* antigen negative and positive internal control preparations were provided by the manufacturer. Results were expressed as absolute 450/605 nm 3,3′,5,5′-Tetramethylbenzidine (TMB) product optical density (OD) values. Stool samples were considered ELISA-positive when the corresponding mean OD value was ≥ to the mean *Cryptosporidium* negative internal sample OD + 0.300 OD. Optical density of positive internal controls was checked for each series of experiments.

The sensitivities, specificities, positive predictive values and negative predictive values were calculated according to Loong [14].

### 2.1. Intra-Assay (Test-Retest) Assay

Intra-assay variability was investigated sequentially on the same day with one ELISA-negative and 2 ELISA-positive clinical stool samples diluted 1:10 in PBS (one sample containing *C. parvum* oocysts and 1 containing *C. chipmunk* genotype oocysts). For each sample, 6 independent ELISA were performed in triplicates (different plates, same reagents).

### 2.2. Inter-Assay ELISA Reproducibility

The replicate (inter-assay) ELISA reproducibility was assessed using one ELISA-negative sample and one ELISA-positive sample (containing *C. hominis* oocysts), tested independently in triplicates on 4 different days.

### 2.3. Statistical Evaluation of Results

Contingency analysis was performed using exact Fisher’s test. Distribution comparisons between series of results were performed using t tests, thus assuming normal-like distribution of data. Correlation trends were estimated by calculating r correlation coefficient values.

## 3. Results and Discussion

### 3.1. Effects of Human Clinical Stool Preservation on Cryptosporidium spp. Antigen ELISA Detection

Most available clinical samples being preserved in K2CRO4 and/or frozen buffers, preliminary experiments were aimed at evaluating the effects of K2CRO4 treatment and freezing on ELISA antigen detection. As shown in Table 1, no difference in OD values was observed between undiluted stool, 1:10 stool dilution in PBS, 1:1 stool dilution in 2.5% K2CRO4 PBS, and undiluted stool kept frozen at (−80 °C) (*p* > 0.05). Specifically, all samples were found antigen positive.

### 3.2. Intra-Assay Variability and Inter-Assay ELISA Reproducibility

For the intra-assay, the mean OD (±SD) were 0.326 (±0.032); 1.210 (±0.108) and 4.108 (±0.385) for oocyst-negative, and stools with *C. parvum* and *C. chipmunk* oocyst, respectively, corresponding to a coefficient of variation lower than 10% for experimental samples. The ELISA reproducibility resulted in mean OD 0.329 (±0.034) and 3.600 (±0.231), respectively.

### 3.3. Influence of Cryptosporidium spp. oocyst Concentration in Clinical Stools on ELISA Antigen Detection

ELISA detection cut-off value for clinical samples was estimated in a pilot study according to *C. parvum* oocyst counts in stools. As shown in Table 2, OD exhibited moderate variation (from 3.690 to 4.407) from <1 oocyst to 76 oocysts/50 MF, which suggests that ELISA may detect antigens in clinical samples containing very low oocyst concentrations and considered microscopically negative.

### 3.4. Inter-Laboratory Evaluation of ELISA Sensitivity and Species Specificity for Cryptosporidium spp. Oocyst-Positive Random Clinical Samples

For 50 oocyst-positive stools separately investigated by Lab#1 and Lab#2 (Table 3), close OD values were obtained by the two laboratories (ranges 0.199–4.420, and 0.184–5.327, means OD (±SD): 3.952(±1.620) and 3.572(±1.303), in Lab#1 and Lab#2, respectively, r = 0.93). Mean cut-off values were 0.496(±0.021) and 0.480(±0.051), for Lab#1 and Lab#2, respectively. Close mean OD values were obtained for the 20 *C. parvum* and the 20 *C. hominis* oocyst-positive stools (4.319(±0.792) and 3.772(±0.146), respectively, *p* > 0.05). All Samples with *C. felis*, *C chipmunk, C. meleagridis* and *C. canis* sample were ELISA positive in both laboratories. Identical decisions on ELISA positivity/negativity were obtained for all samples with 2/50 exceptions, i.e., one *C. hominis*-PCR positive sample, for which cut-off values (0.474 and 0.594, respectively) resulted in discrepant negative and positive conclusion, respectively. Two *C. cuniculus*-PCR positive samples exhibited low OD and were considered negative by both laboratories.

As summarized in Table 4, ELISA detection of *Cryptosporidium* spp. antigens in 176 oocyst-negative clinical samples resulted in 166 true negative and 10 false-positive results

Among the negative controls, 6 and 1 were found ELISA-positive (OD range: 0.671–3.549) by Lab#1 and Lab#2, respectively. In Lab#1, 4/6 samples were controlled negative by microscopic examination, but positive by PCR. The false-positive sample found by Lab#2 was positive for *strongyloides stercoralis*. At first, it was suspected cross-contamination between *Cryptosporidium* and nematode antigens, but this was ruled out as *Strongyloides stercoralis* ova found in few other samples were negative with the ELISA. Lab#3 found three false-positive samples that, after microscopy and PCR control, turn out to be negative to Cryptosporidium. No other intestinal parasites were found during this control.

## 4. Conclusions

In summary, ELISA detection of *Cryptosporidium* spp. coproantigens performed in 88 oocyst microscopy-positive clinical samples and 176 microscopy-negative samples resulted in 87 true positive, a false negative, 166 true negative, and 10 false negative results in at least one lab leading to sensitivity, specificity, positive and negative predictive value of 98.86%, 94.32%, 89.69%, and 99.40%, respectively. These data show that the method is efficient to identify *Cryptosporidium* oocysts in stool samples whatever the species with the exception of *C. cuniculus,* which seems to give lower OD values, but this result has to be confirmed as few samples were evaluated. Interestingly, the quantity of oocysts did not interfere with the result as sample with few or many oocysts were detected with similar OD values as previously described [8].

Coproantigen detection using ELISA method requires minimal training thus appeared to be easy to perform, as well as accurate for epidemiological studies and diagnostic purposes of *Cryptosporidium* infection, compared with conventional microscopic methods.

## Figures and Tables

**Table 1:** Influence of PBS dilution, K_2_Cr_2_O_7_ addition and freezing of *Cryptosporidium* oocyst-positive samples on ELISA optical density (OD) values.

Stool Sample #	Undiluted	1:10 Dilution in PBS	1:1 Dilution in 2.5% K_2_Cr_2_O_7_ PBS	Frozen (−80 °C)
1	4.407	4.345	4.242	4.030
2	4.296	4.249	4.294	4.328
3	4.399	4.258	4.325	4.600
4	4.333	3.690	4.416	4.474
5	4.471	4.443	4.292	4.325

Results expressed as 405/605 nm mean OD values of triplicates. The coefficient of variation (CV), which is the measure of relative variability; the ratio of the standard deviation (SD) to the mean, accounted for less than 10%. #: sample number.

**Table 2:** Influence of stool oocyst concentration on ELISA antigen detection.

Sample #	Oocyst Concentration in Undiluted Stool (Number/50 MF)	OD	Oocyst Concentration in 1/10 PBS Diluted Stool (Number/50 MF)	OD
1	76	4.407	13	4.345
2	23	4.296	6	4.249
3	17	4.471	4	4.443
4	5–10	2.743	NA	NA
5	5–10	4.375	NA	NA
6	6	4.399	1	4.258
7	4	4.333	<1	3.690

Results expressed as 405/605 nm mean OD values of triplicates. The coefficient of variation accounted for less than 10%. #: sample number; MF: microscopic fields; NA: non-available.

**Table 3:** Summary of ELISA results for *Cryptosporidium* spp. oocyst positive stool samples.

	Lab#1 ELISA Positive Samples	Lab#2 ELISA Positive Samples	Labs #1 and #2 Consensus ELISA Results	Lab#3	Lab#1: Additional Positive Samples	Oocyst and ELISA Positive Samples
ELISA Positive (n)	49	47	47	28	12	87
Total (n)	50	50	48	28	12	88

All 12 stool *Cryptosporidium parvum*-PCR positive samples studied separately by Lab#1 were ELISA positive (mean OD 3.470 ± 1.201, mean cut-off OD 0.462 ± 0.034). For 28 *C. parvum* PCR-positive random samples examined by Lab#3, OD ranged from 0.800 to 4.060 (mean cut off 0.650 ± 0.035), and all were considered ELISA-positive.

**Table 4:** Summary of ELISA results for *Cryptosporidium* spp. oocyst negative samples.

	Lab#1	Lab#2	Lab#3	Total
ELISA negative	50	59	57	166
ELISA positive	6	1	3	10
Total	56	60	60	176

## Data Availability

Not applicable.

## Appendix A

*French Cryptosporidiosis Network*

Isabelle Accoceberry, Adela Angoulvant, Nicolas Argy, Dominique Aubert, Patrick Bastien, Ghania Belkadi, Antoine Berry, Denis Blanchet, Julie Bonhomme, Françoise Botterel, Marie Elizabeth Bougnoux, Julie Brunet, Gabriela Certad, Cathy Chemla, Eric Dannaoui, Marie Laure Darde, Anne Debourgogne, Luc de Gentile, Brigitte Degeilh, Pascal Delaunay, Nicole Desbois, Guillaume Desoubeaux, Pierre Flori, Emilie Frealle, Cécile Garnaud, Frédéric Grenouillet, Samia Hamane, Sandrine Houze, Franck. Labbé, Denis Leméteil, Coralie Lollivier, Yohann Le Govic, Denis Magne, Pierre Marty, Jean Menotti, Florent Morio, Gilles Nevez, Muriel Nicolas, Philippe Poirier, Christelle Pomares, Meja Rabodonirina, Florence Robert Gangneux, Marie Hélène Rodier, Milene Sasso, Marc Thellier, Anne Totet, Stéphane Valot, Odile Villard, Isabelle Villena, Hélène Yera.
